# New support for an old hypothesis: density affects extra-pair paternity

**DOI:** 10.1002/ece3.489

**Published:** 2013-02-13

**Authors:** Christian Mayer, Gilberto Pasinelli

**Affiliations:** 1 University of Zurich, Institute of Evolutionary Biology and Environmental StudiesWinterthurerstrasse 190, Zurich, Switzerland, CH-8057; 2 FORNAT AGUniversitätstrasse 65, Zurich, Switzerland, CH-8006; 3 Swiss Ornithological InstituteSeerose 1, Sempach, Switzerland, CH-6204

**Keywords:** Birds, density, extra-pair parentage, microsatellites, population

## Abstract

Density has been suggested to affect variation in extra-pair paternity (EPP) in avian mating systems, because increasing density promotes encounter rates and thus mating opportunities. However, the significance of density affecting EPP variation in intra- and interspecific comparisons has remained controversial, with more support from intraspecific comparisons. Neither experimental nor empirical studies have consistently provided support for the density hypothesis. Testing the density hypothesis is challenging because density measures may not necessarily reflect extra-pair mating opportunities, mate guarding efforts may covary with density, populations studied may differ in migratory behavior and/or climatic conditions, and variation in density may be insufficient. Accounting for these potentially confounding factors, we tested whether EPP rates within and among subpopulations of the reed bunting (*Emberiza schoeniclus*) were related to density. Our analyses were based on data from 13 subpopulations studied over 4 years. Overall, 56.4% of totally 181 broods contained at least one extra-pair young (EPY) and 37.1% of totally 669 young were of extra-pair origin. Roughly 90% of the extra-pair fathers were from the adjacent territory or from the territory after the next one. Within subpopulations, the proportion of EPY in broods was positively related to local breeding density. Similarly, among subpopulations, proportion of EPY was positively associated with population density. EPP was absent in subpopulations consisting of single breeding pairs, that is, without extra-pair mating opportunities. Our study confirms that density is an important biological factor, which significantly influences the amount of EPP within and among subpopulations, but also suggests that other mechanisms influence EPP beyond the variation explained by density.

## Introduction

Despite considerable efforts, the underlying factors determining variation in levels of extra-pair paternity (EPP) among species and among populations of the same species are still not fully understood. In particular, there is still debate about the influence of population-specific ecological factors (e.g., density and synchrony) on levels of EPP. Variation in population density is one of the classic factors proposed to explain inter- and intraspecific variation in EPP in avian mating systems. The density hypothesis states that increased proximity among individuals increases encounter rates and mating opportunities when searching for potential extra-pair mates, thereby reducing the costs of extra-pair matings. Thus, if density increases, the rate of EPP should increase as well (Westneat et al. 1990). However, the density hypothesis has fallen into disfavor because there is moderate evidence for a general relationship between population density and EPP across species (Griffith et al. 2002; but see Westneat and Sherman 1997; Moller and Ninni 1998). At the intraspecific level, an effect of density on EPP has been shown in experimental studies (Gowaty and Bridges 1991; Charmantier and Perret 2004; Stewart et al. 2010) and in some observational studies (e.g., Gibbs et al. 1990; Yezerinac et al. 1999; Ryder et al. 2012), but not in others (see Appendix S2).

Assessing the relationship between density and EPP rate in nonexperimental studies is challenging for various reasons (Griffith et al. 2002). One of the challenges is to choose a density measure that potentially reflects extra-pair mating opportunities. If extra-pair copulations (EPC) mainly occur in the area around a territory (Bouwman et al. 2006), then a measure of local breeding density reflects extra-pair mating opportunities and is likely to be linked to EPP rates. In contrast, if EPCs take place well beyond the immediate territory neighborhood, and males and females encounter each other at common sites (e.g., Dunn et al. 1994b; Reyer et al. 1997), local breeding density or territory structure is unlikely to be related to variation in EPP. The rate of EPP might also be decoupled from local breeding density if non-territorial floater males are common (Tarof et al. 1998; Ewen et al. 1999) or if the species is not territorial (Griffith et al. 1999; Westneat and Stewart 2003; Dunn and Whittingham 2007).

Another reason for the difficulty of assessing the relationship between population density and EPP rate is that mate-guarding efforts may increase at high density. Social males may invest more effort preventing extra-pair matings of their females at increased densities (Komdeur 2001). In this case, mate guarding could compensate for a density-dependent increase in opportunity for EPP (Kokko and Rankin 2006). Along the same lines, mate-guarding efforts may be more effective if more crowded habitats are visually less occluded; thereby allowing males to more successfully prevent extra-pair encounters of their social females. The potentially confounding effect of habitat structure on mate-guarding success may be strong only when comparing across populations (Westneat and Mays 2005).

A third reason potentially obscuring the relation between density and EPP rate is a difference in migration distances among the populations studied. The reasoning is that long migration distances increase the need to settle quickly resulting in inaccurate or hasty mate choice. As a consequence, the proportion of high quality females paired to low quality males may increase, which enhances the benefits to females of pursuing EPCs (Weatherhead and Yezerinac 1998). Long migration distance may thus increase the level of EPP in populations at higher latitudes (Spottiswoode and Moller 2004) and could therefore obscure the effect of density on EPP when populations at different latitudes are compared.

Finally, variation in local breeding density may be insufficient to find an effect on EPP. A relationship between density and EPP is not predicted if density exceeds a threshold resulting in sufficient extra-pair partners at all local densities (Westneat et al. 1990). Similarly, a relationship between density and EPP should not occur when densities are so low that potential extra-pair mates do not encounter one another (Orell et al. 1997).

Many studies addressing the density hypothesis compared differences in EPP rates between individuals within the same population, and the few studies on EPP in relation to density across populations involved a small number of populations (Griffith et al. 2002). Here, we present data on density and EPP rates from multiple wetland fragments hosting subpopulations of the reed bunting (*Emberiza schoeniclus*) in Switzerland. We tested two predictions of the density hypothesis. First, we predicted that levels of EPP within subpopulations were positively related to local breeding density, assessed through measures at the territory level. Second, we expected that levels of EPP among subpopulations were positively related to breeding density, assessed at the level of the subpopulation.

The reed bunting is a small socially monogamous short distance migrant restricted to wetlands (Glutz von Blotzheim and Bauer 1997). High levels of extra-pair paternity (up to 55% extra-pair young in 86% of broods) have been reported from populations throughout Europe (Dixon et al. 1994; Bouwman et al. 2005; Kleven and Lifjeld 2005; Suter et al. 2007). The reed bunting defends only nesting territories (Glutz von Blotzheim and Bauer 1997). Both sexes forage outside these territories. In spite of this, EPP has been shown to mainly occur among close neighbors (Bouwman and Komdeur 2006; Bouwman et al. 2006) and floaters apparently are rare (own observation). Consequently, density estimates at the level of the territory likely reflect and hence should correlate with extra-pair mating opportunities. Adults forage in open habitat (Marthinsen et al. 2005) and nest cryptically within old, rather dense reed beds (*Phragmites* sp.) (Pasinelli and Schiegg 2006), where vision is frequently obstructed. Reed bunting mate-guarding efforts do not vary with density (Marthinsen et al. 2005). In our study, then, neither habitat structure nor mate-guarding efforts are likely to vary with density potentially masking a density-dependent response in EPP rate. The subpopulations studied are scattered within an area of 200 km^2^ in the Swiss lowlands; hence, any potential effect of migration distance on EPP variation among populations is negligible. Similarly, additional confounding factors possibly arising from individuals with different behavioral and ecological backgrounds sampled in sites far apart were accounted by assessing EPP rates in subpopulations across a comparatively small area. Numbers of breeding pairs in the subpopulations studied ranged from 1 to 50, and accordingly, variation in breeding density among populations was high. Thus, both the study species and the study setup seem to be appropriate to confront the challenges outlined above.

## Material and Methods

### Field work

**Figure 1:**
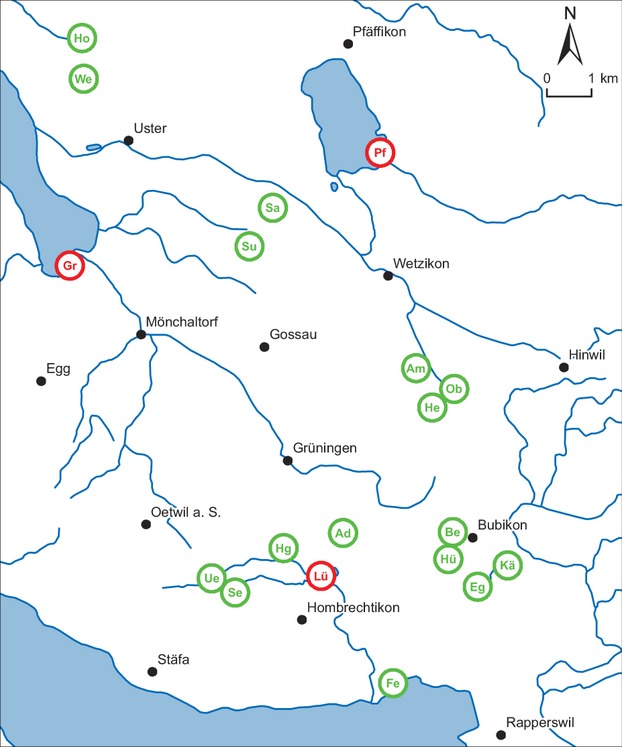
Location of the subpopulations studied in northeastern Switzerland. Red circles indicate the three large subpopulations, green circles the small subpopulations. Letters inside circles represent the first two letters of the subpopulation names shown in Table 1. Source: Federal Office of Topography.

The study was carried out in wetland nature reserves scattered over an area of 200 km^2^ in south-eastern Canton Zurich, Switzerland, from 2002 to 2005. The reserves range in size from 1.9 to 247.2 ha (median 10.5 ha, interquartile range 4.2–16.7 ha) and represent all potentially suitable breeding localities for reed buntings within this region (Table 1, Fig. 1). The limits of each wetland reserve were based on vegetation data taken from the land use maps of the Cantonal Office for Nature Conservation. We defined as a subpopulation the breeding pairs within each wetland reserve. In the three largest subpopulations (circled in red in Fig. 1), 20–60 pairs of reed buntings bred annually (Orniplan, unpubl. report; G. Pasinelli, unpubl. data). Here, reproductive performance of at least 10 breeding pairs per subpopulation was annually monitored in randomly selected study plots along the lakefront. The study plots had been selected at the beginning of the study in 2002, and the same plots were monitored in all years. In the other 16 subpopulations, all breeding pairs present were annually monitored, with the annual number of breeding pairs ranging from 0 to 5.

**Table 1:** Overview on the subpopulations studied from 2002 to 2005

Subpopulation	Coordinates	Size (ha)	Old reed area (ha)	Breeding pairs	Broods	Offspring	DNA	BP	SFU	PF
Adletshausen	47^o^16′/08^o^47′	4.2	0–0.022	0.25	2	7	2/7			
Ambitzgi^a^	47^o^18′/08^o^48′	16.7	0–0.543	0.25						
Bergli	47^o^16′/08^o^48′	5.6	0.300–0.356	1.75	10	33	2/6			
Egelsee	47^o^15′/08^o^49′	16.3	0.059–0.559	2.25	10	44				
Feldbach	47^o^14′/08^o^48′	2.7	0.383	2	7	25				
Greifensee	47^o^19′/08^o^42′	44.1	0.972–1.382	12	46	168			2/8	
Hellberg	47^o^18′/08^o^48′	1.9	0–0.096	0.5	2	9		2/9		
Herrgass	47^o^16′/08^o^46′	2.4	0.181	0.25	1	4		1/4		
Hopperen	47^o^22′/08^o^42′	8.7	0.244–0.376	0.75	1	4				
Hüsli	47^o^16′/08^o^49′	14.0	0.133	2.25	11	35		1/4		
Kämmoos	47^o^16′/08^o^50′	10.5	0.028–0.413	1.25	8	25		3/8		
Lützelsee	47^o^16′/08^o^47′	54.7	1.314–1.812	12	43	171			3/13	
Oberhöfler	47^o^18′/08^o^48′	38.5	0.201	0.5	3	10		3/10		
Pfäffikersee	47^o^21′/08^o^47′	247.2	2.581	10.25	43	155			3/10	
Sackried	47^o^21′/08^o^45′	5.7	0.522–0.881	1.25	5	21		4/16		
Seeweidsee	47^o^16′/08^o^47′	5.2	0.364	1.5	5	20		2/8		
Sulzbach	47^o^15′/08^o^45′	2.9	0.195	0.75	3	14		3/14		
Uerzikon	47^o^15′/08^o^45′	10.9	0.478	3.75	9	28				2/8
Werrikon	47^o^22′/08^o^42′	13.0	0.626–0.853	2.75	6	24				1/3
Total					215	797	4/13	19/73	8/31	3/11

Size based on wetland censuses of the canton of Zurich in 1976/77; old reed area based on own censuses with GPS and referring to area actually monitored in the three large subpopulations (Greifensee, Lützelsee, Pfäffikersee) and to the entire wetland (in the other subpopulations), respectively. Note that old reed area may vary among years as a consequence of wetland management. Breeding pairs gives the mean annual number of breeding pairs per subpopulation. Broods = number of broods from which blood samples were obtained from all offspring. The last four columns refer to the number of broods (before the back slash) and to number of nestlings excluded from the data set, with the column headings indicating the reasons for exclusion: DNA = insufficient DNA quality, BP = only 1 BP per year present, SFU = social father unknown, PF = polygynous father. Further explanations are found in the chapter “Dataset preparation”.

a No genetic data available, as nest was lost to predation.

**Figure 2:**
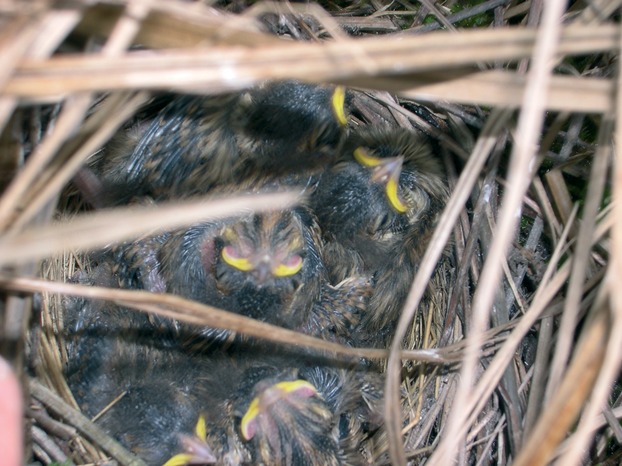
Reed bunting nestlings approx. 8 days old in northeastern Switzerland, 19 May 2005. Picture: G. Pasinelli.

Monitoring of reproduction took place from early March, when males return from their wintering grounds, to early August, when the breeding period ends. In our study area, reed bunting males established territories in old reed habitat, in which most nests were built by the females (see Pasinelli et al. 2008, 2011 for details). Nests were located using behavioral cues, including nest building and parental visitation patterns, during incubation and nestling care. The young were banded between nestling day 6 and 9 (Fig. 2), with each nestling obtaining a numbered aluminum ring and a unique combination of three colored plastic rings allowing individual identification in the field. After fledging or nest loss, nest locations were recorded using a hand-held global position system (GPS) receiver (GPS-12XL with RXMAR decoder, Garmin, Olathe, KS; GeoExplorer 3, Trimble, Sunnyvale CA; Leica GS50, Leica, St. Gallen, Switzerland). The precision of the GPS locations after differential correction was ≤2 m. Adult males were captured with mist nests either by luring them with a song playback in March and April or by placing the nets at least 2 m from the nest when nestlings were fed. The latter approach was also used to capture adult females. Adults were color-marked in the same way as nestlings, and social parents were determined by observation of color-ringed individuals during nest building, incubation and the nestling period. Each breeding pair was observed at least twice a week. At the time of banding, we collected DNA samples of adults and nestlings by puncturing the brachial vein and absorbing blood (max. 100 μL) with heparinized microcapillaries (permission number from the Cantonal Veterinary Office Zurich: 169/2001). Samples were either stored in microcapillaries directly or blown into APS-buffer (Arctander 1988) and stored at −20°C. We also collected dead nestlings and eggs that failed to hatch and stored them at −20°C for later DNA extraction.

### Laboratory work

DNA from blood, unhatched eggs, and dead nestlings was extracted with the “Biosprint 96 DNA Blood Kit” from Qiagen AG (Hombrechtikon, Switzerland). As characterized in Mayer et al. (2008), we used 10 autosomal microsatellite loci (Emb 03, Emb 07, Emb 12, Emb 17, Emb 19, Emb 27, Emb 79, Emb 81, Emb 89, Emb 90, and Emb 112) and four additional z-linked microsatellite loci (Emb 79, Emb 84, Emb 107, and Emb 117) for parentage analyses. Polymerase chain reaction amplification was conducted as described in Mayer et al. (2008). Amplified fragments were visualized on an ABI PRISM 3730 Avant capillary sequencer. Allele sizes were determined in relation to an internal size standard (GeneScan-500LIZ) using GENEMAPPER version 3.7. Details on number of alleles, heterozygosity, tests of Hardy–Weinberg equilibrium and presence of null alleles for the markers employed here can be found in Mayer et al. (2009).

### Parentage analysis

Based on the 10 autosomal microsatellite loci, parentage was determined in three steps using a likelihood-based approach in CERVUS 2.0 (Marshall et al. 1998). For all steps, the program screened candidate individuals and ranked them by the likelihood of being the nestling's parent. First, maternity was determined for each nestling to check for egg dumping. This step included 232 broods. The genetic mother was identified in 208 broods, and no egg dumping was detected in these cases because the social mother always corresponded to the genetic mother. For the remaining 24 broods, we did not have the genotype of the social mother. In the second step, paternity was assigned for the 208 nests using the mother as “known parent” in the analysis. The 10 autosomal microsatellite loci had a combined exclusionary power of 0.99984 for the first parent and 0.9999984 for the second parent. Finally, as we did not have the genotype of the social mother for 24 nests, we determined paternity for those nests in a separate analysis without the genetic information of the social mother. The exclusionary power was reduced in those cases, multiple candidate fathers carrying common genotypes may have remained unexcluded. To increase the certainty of paternity, we added information of the four sex-specific z-linked microsatellite loci and manually checked for congruence between offspring, their social fathers and the candidate father's genotypes. We did the same when nestlings did not amplify at all autosomal loci. Samples with bad DNA quality, that is, which did not amplify at more than four autosomal loci, were excluded.

In cases where the social father, or the best candidate father, mismatched with the offspring genotype, we checked the raw data for editing and typing errors. Seventeen nestlings mismatched at one locus with their potential genetic fathers. However, in all those cases no alternative candidate males had an almost similarly high likelihood of being the genetic father. When we compared those nestlings to their potential genetic fathers at the four z-linked loci, no mismatches could be detected. We therefore propose that the 17 mismatches arose from mutation. If we assume that highly polymorphic microsatellites mutate at the rate of 10^−3^ (Weber and Wong 1993; Balloux and Lugon-Moulin 2002), the number of observed mismatches is consistent with 16 mutations expected for our dataset (1171 individuals × 14 loci × 10^−3^).

### Dataset preparation

Initially, we monitored reproduction of the reed bunting in 19 subpopulations (Table 1). A number of nests had to be excluded (for various reasons outlined below), leaving us for statistical analysis with a total of 669 nestlings from 181 broods in 13 subpopulations collected over 4 years. There were no nestling data for one of the initial 19 subpopulations (Ambitzgi), because the single nest within this subpopulation was lost to predation. We excluded some nests from the dataset before testing the effect of density on EPP for the following reasons. First, we excluded data of four nests (13 nestlings) because nestling DNA quality was insufficient to allow reliable paternity analysis. Two of those nests were the only nests produced in the subpopulation Adletshausen (Table 1). Therefore, the exclusion of those nests reduced the number of subpopulations to 17. Second, we excluded 19 broods (73 nestlings) from subpopulations where only a single pair was breeding, because the density hypothesis requires that adults have the opportunity to encounter extra-pair mates. As revealed by radio-tracking, breeding adults in our study area did not leave their subpopulations during the breeding season (Silvestri 2006). Third, we excluded broods where the genotype of the genetic and the social father remain unknown (social father not captured). In those cases, it was impossible to determine whether nestlings were sired by the social father or an unknown extra-pair male (8 broods with 31 nestlings). Finally, we excluded three broods (11 nestlings) from two polygynous males. Polygyny can have a strong influence on paternity (Rätti et al. 2001), as polygynous males have, but cannot guard, more than one female at the same time. Polygynous males may therefore more likely to be cuckolded in comparison to their socially monogamous neighbors (Birkhead and Møller 1992). At the same time, polygyny could enable later arriving females to choose an attractive male, making it unnecessary for those females to adjust their initial mate choice by pursuing extra-pair fertilizations. Thus, polygyny could also decrease the frequency of extra-pair fertilizations (Hasselquist et al. 1995).

### Density estimation

We generated two measures of local breeding density at the level of the pair (i.e., within subpopulations): (1) distance to nearest reed bunting territory in meters (hereafter “nearest neighbor distance”); and (2) number of territories within 170 m of the center of the focal territory (hereafter “number of neighbors”). Territory centers were defined as the geometric mean of all nests produced per territory per year. The radius of 170 m around a territory corresponds to the average distance between territory centers of extra-pair males and the males they cuckolded within subpopulations of our study area. While the nearest neighbor distance only takes the distance to the next possible extra-pair partner into account, the number of territories within 170 m reflects the number of extra-pair mating opportunities within the neighborhood of a focal territory.

For comparisons among subpopulations, we calculated subpopulation specific measures of density as (1) the median nearest neighbor distance; and (2) the median number of neighbors within 170 m, respectively, for each subpopulation. These calculations were performed in ArcGIS 9.3. Additionally, we calculated density as the number of territories in old reed habitat divided by the extent of old reed habitat (ha) per subpopulation. This yielded an estimate of (sub-)population density per ha, which has commonly been used in tests of the density hypothesis (hereafter ‘density per ha’). Old reed is a key habitat for the reed bunting when settling in early spring after migration (Surmacki 2004) and affects the number of territories per study subpopulation. We recorded old reed area annually with GPS.

### Data analysis

To test for the relationship between density and EPP rate, we used generalized linear mixed models with a logit link and binomial errors as implemented in the lmer procedure of the lme4 library, a contributed package to the open source statistical software R (R Development Core Team 2012). We first tested for the effect of density on EPP within subpopulations. This analysis comprised EPP data of broods of all subpopulations with annually more than one breeding pair collectively. The response variable was the EPP rate in a brood (i.e., extra-pair young to total number of young per brood). Explanatory variables were the local breeding density as fixed effect and subpopulation identity, the subpopulation-by-density interaction, year, and female identity nested within subpopulation as random effects. As the two measures of local breeding density, the nearest neighbor distance and the number of neighbors, were highly correlated (Spearman rank correlation *r*_s_ = −0.707, *n* = 181, *P* < 0.001), we tested for their effects on EPP rate separately. The random effect subpopulation-by-density interaction was included to test whether a potential relationship between density and EPP rate may differ among subpopulations. A random factor subpopulation identity was included in the model to estimate the variance in EPP that is generated due to specific characteristics (e.g., habitat structure) of subpopulations. Year and female identity (the latter nested within subpopulation) were included in the model to account for the variance in EPP levels generated by the effects of years and individual females' propensities to seek EPC. Female identity also accounted for dependencies arising from the use of data from multiple nests of the same female within and between seasons. We tested for significance of random effects with likelihood-ratio tests for nested models. Here, the full model is compared to a reduced model without the random effect to be tested.

To test for relations between population density and EPP among subpopulations, we analyzed models with EPP rate per subpopulation (i.e., extra-pair young to total number of young per subpopulation) as response variable and density (fixed effect), subpopulation identity and year (random effects) as explanatory variables. As the two measures of local breeding density were again highly correlated (Spearman rank correlation *r*_s_ = −0.681, *n* = 34, *P* < 0.001), we tested for their effects on EPP rate per subpopulation separately. Finally, we assessed the relationship between population density and EPP among subpopulations with a model identical in terms of the response variable and the random effects as just explained, but using density per ha (fixed effect) as explanatory variable instead of the measures of local breeding density. In all among-subpopulation analyses, we avoided pseudoreplication using only one randomly selected brood for the 45 females that produced two or more broods within a given year and subpopulation.

## Results

### Paternity

Hundred-and-two broods (out of totally 181 broods from 13 subpopulations) contained at least one extra-pair young (EPY) (56.4%) and 248 nestlings (out of totally 669) were EPY (37.1%). Across subpopulations and years, extra-pair paternity rate ranged from 0 to 0.75 (Appendix S1, Supporting information). We identified 120 extra-pair fathers of which 23 (19.2%) had an unknown genotype (i.e., were not among the banded males). For nine extra-pair fathers with known genotype the location of their territory remained unknown. Three of them were banded after the year in which they sired extra-pair young, so that we were not able to locate their territory in the relevant year. The other six genotyped extra-pair fathers with unknown territories occurred in the three large populations, which comprised more breeding pairs than we were able to monitor. Of the 88 extra-pair fathers, for which both genotype and territory location was known, 68.2% were direct neighbors (adjacent territory), and 21.6% were close neighbors (one territory in between) to the territories in which they sired EPY. Except for one male siring three nestlings within a brood of a neighboring subpopulation at approx. 500 m distance to his own territory, extra-pair males exclusively sired EPY within subpopulations. Subpopulations occupied by single breeding pairs in a given year exclusively contained within-pair young (60 nestlings of 16 broods). Those nestlings were not included in the following analyses.

### Relationship between density and extra-pair paternity

Extra-pair paternity was significantly related to both measures of local breeding density within subpopulations, negatively to the nearest neighbor distance and positively to the number of neighbors (Table 2). The density-by-subpopulation interaction was not significant (Table 2), indicating that there was a consistent relationship between EPP rate and local density within all subpopulations. The random factor year was significant, pointing at differences in EPP rate across years (Table 2, Appendix S1). Variation in levels of EPP within subpopulations was high, and female identity always explained a significant amount of the overall variance in EPP rate (Table 2).

**Table 2:** Summary of generalized linear mixed models testing the relationships between density and extra-pair paternity rates in the reed bunting within and among subpopulations

	Within subpopulations			Among subpopulations		
		
Effect	Estimate	Test statistic	*P*	Estimate	Test statistic	*P*
A) Nearest neighbor distance						
NND	−0.007 (0.003)	−2.147	0.032	−0.007 (0.003)	−2.097	0.036
Subpopulation	0.000 (0.000)	0.285	0.593	0.187 (0.432)	1.54	0.215
Subpopulation x NND	0.000 (0.000)	0.000	0.999			
Year	0.178 (0.442)	4.382	0.036	0.015 (0.123)	0.248	0.619
Female	2.863 (1.692)	73.977	< 0.001			
B) Number of neighbors						
NN	0.274 (0.066)	4.179	< 0.001	0.207 (0.044)	4.674	0.007
Subpopulation	0.000 (0.000)	1.002	0.317	0.000 (0.000)	0.000	1
Subpopulation x NN	0.000 (0.000)	0.000	1			
Year	0.256 (0.506)	7.349	0.007	0.021 (0.146)	0.52	0.471
Female	2.289 (1.513)	58.833	< 0.001			

In within-subpopulation analyses, fixed factors were local breeding density estimated as the nearest neighbor distance and the number of neighbors, respectively. Random factors were subpopulation identity, the subpopulation identity-by-density interaction, year, and female identity nested within subpopulation. In among-subpopulation analyses, fixed factors were density estimated as the median nearest-neigbor distance and the median number of neighbors, respectively. Random factors were subpopulation identity and year. For fixed effects, parameter estimates with standard errors (in parentheses), z-values and *P*-values are given. For random effects, variance components with (standard deviation) as well as χ^2^ values and *P*-values of likelihood-ratio tests are given. Data from 181 broods collected in 13 subpopulations over 4 years.

NND = nearest neighbor distance, NN = number of neighbors.

**Figure 3:**
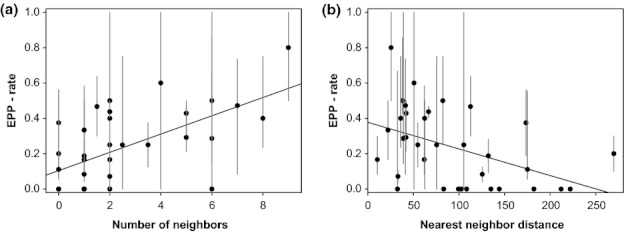
Extra-pair paternity rate per subpopulation and year in relation to a) the median number of neighbors and b) the median nearest neighbor distance (m) per subpopulation and year. Each filled circle represents the median of the EPP rate within a subpopulation in a specific year. Lines (interquartile range) show the variation in EPP rates among territories within subpopulations. *N* = 181 broods from 13 subpopulations collected over 4 years.

EPP rate at the subpopulation level was positively related to population density measured as the median number of neighbors. Conversely, EPP rate at the subpopulation level was negatively related to population density measured as the median nearest neighbor distance (Table 2, Fig. 3). Variation in EPP rate did not differ among subpopulations or years (Table 2). Finally, EPP rate at the subpopulation level was not significantly related to population density measured as density per ha (estimate ± SE = 0.048 ± 0.027, z = 1.75, *P* = 0.081, *n* = 34).

## Discussion

Results of this study conducted with data from 13 subpopulations are consistent with the density hypothesis. Local density at the territory level and population density at the subpopulation level significantly explained variation in EPP rate of reed buntings.

### Within-population studies

Most previous tests of the density hypothesis were carried out at the within-population level, and many of these tests suggested that density was a relevant factor explaining variation in EPP (e.g., Gowaty and Bridges 1991; Moller 1991; Hasselquist et al. 1995; Gray 1996; Bjornstad and Lifjeld 1997; Hoi and Hoi-Leitner 1997; Westneat and Sherman 1997; Langefors et al. 1998; Moller and Ninni 1998; Richardson and Burke 1999; Charmantier and Perret 2004; Estep et al. 2005; Lindstedt et al. 2007; Stewart et al. 2010; Ryder et al. 2012). Our study corroborates these within-population analyses. However, within-population tests of the density hypothesis are vulnerable to methodological or interpretation problems. For example, many of the studies that did not find support for the density hypothesis within populations suspected that their estimates of local breeding density did not reflect extra-pair mating opportunities, because a large proportion of EPCs occurred away from the territories used to determine local breeding density (Dunn et al. 1994a; Reyer et al. 1997; Moore et al. 1999; Westneat and Mays 2005). In other studies, males were not territorial at least at the time when pursuing EPCs (Bollinger and Gavin 1991; Hill et al. 1994; Barber et al. 1996; Rätti et al. 2001), which decoupled breeding density from extra-pair mating opportunities. When density estimates do not reflect extra-pair mating opportunities, the within-population approach is likely to be inappropriate to address the density hypothesis because extra-pair mating opportunity, rather than density per se, is the mechanism underlying the hypothesis (Westneat et al. 1990). In our study, the vast majority of males siring EPY were close territorial breeding neighbors and thus our measures of local density most likely reflected extra-pair mating opportunities. In another study on the reed bunting, only a positive trend between the proportion of EPP and local breeding density was found, even though the majority of males siring EPY again were close territorial breeding neighbors (Bouwman and Komdeur 2006). Bouwman and Komdeur (2006) suggested that mate-guarding efforts increased with cuckoldry risk at increasing density (Komdeur 2001; Estep et al. 2005), thereby masking the effect of density on EPP. This has also been assumed as explanation of the lacking relationship between density and EPP in other studies (Thusius et al. 2001; Westneat and Mays 2005). However, reed bunting mate-guarding efforts do not appear to vary with density (Marthinsen et al. 2005).

As an alternative explanation of the insignificant relationship between EPP and local breeding density, Bouwman and Komdeur (2006) suggested that local breeding density may have exceeded a threshold resulting in sufficient extra-pair mating partners at all densities. A density threshold may also have obscured a relationship between density and EPP in other studies (Dunn et al. 1994b; Tarof et al. 1998; Johannessen et al. 2005). However, unambiguous support for the ‘threshold hypothesis’ initially proposed by Westneat et al. (1990) is still lacking. In our study, nearest neighbor distances varied from 10 to 270 m, and the number of neighbors within 170 m of the focal territory varied from 0 to 11. Bouwman and Komdeur (2006) did not report how local breeding densities varied in their population, so we can only speculate that variation in breeding density was sufficient for detecting a significant relationship between density and EPP in our study, but perhaps not in theirs.

A ‘threshold’ may also occur, if local densities are too low, resulting in insufficient extra-pair mating opportunities for all individuals within a population. So far, this situation has been suggested only once (Orell et al. 1997). That no EPP occurred in our subpopulations settled by a single breeding pair may be considered as a manifestation of the postulated low-density threshold.

### Among-population studies

An among-population approach to test predictions of the density hypothesis has been surprisingly rarely applied. Four of them supported the density hypothesis (Gibbs et al. 1990; Yezerinac et al. 1999; Krokene and Lifjeld 2000; Stewart et al. 2010), and three did not (Charmantier and Blondel 2003; Moore et al. 2012; Ryder et al. 2012).

Factors such as migration distance (Spottiswoode and Moller 2004), climate (Bouwman and Komdeur 2006), or habitat (Westneat and Mays 2005) have been shown to influence EPP rate within populations. It is conceivable that these factors may also confound comparisons of EPP rate across populations. In our study, differences among subpopulations in migration distance or climatic conditions were very unlikely given the relatively small study area. Furthermore, we explicitly modeled the potential importance of unknown confounding factors, such as differences in habitat structure or breeding synchrony among subpopulations, including subpopulation identity as a random factor, which, however, turned out to be nonsignificant.

### Which density estimate reflects extra-pair mating opportunities best?

Various density estimates have been used as proxies for extra-pair mating opportunities, but most of these estimates have important shortcomings. For example, the nearest neighbor distance used in our study does not distinguish between situations, where an individual has only one or multiple neighbors. Even though the nearest neighbor distance was negatively related to both estimates of local breeding density and population density based on the number of neighbors, EPP rate was consistently less strongly related to nearest distance than to the number of neighbors (Table 2). Westneat et al. (1990) argued that the number of adjacent neighbors affects the likelihood that individuals seek extra-pair mates and thus captures extra-pair mating opportunities better than the nearest neighbor distance. Consistent with this, Charmantier and Perret (2004) showed in blue tits that the nearest neighbor distance had an effect on EPP rate when the number of neighbors was low, but not when the number of neighbors was high. Stewart et al. (2010) found significant relationships of EPP rate with number of breeding neighbors within 320 m, but not with proximity of the nearest neighbor.

On the other hand, EPP rate among subpopulations was only marginally related to density per ha in our study. Estimating density as the number of territories in relation to the size of the study area is widespread, but may not reflect extra-pair opportunities and may thus not allow an adequate assessment of the density hypothesis. We recommend that the choice of density estimate in studies testing the density hypothesis should be guided by careful consideration of the species' social system and spacing behavior to avoid uninformative results.

### Biological significance of density as a constraint to extra-pair paternity

The idea behind the density hypothesis is compelling. Density affects behavior because it permits increasing interactions between individuals when proximity to or the number of neighbors increases (Westneat and Sherman 1997). Sexual interactions, such as EPCs, seem to be especially sensitive to density, as increased density provides better opportunities to decrease the costs of finding an additional mate (Westneat et al. 1990). Reduced costs of seeking EPC may be one benefit of increased density to both males and females. In females, increased density may additionally allow improved assessment of potential extra-pair mates. The number of potential extra-pair mates and hence the opportunities to engage in EPC with a high quality male likely increase with density leading to increased EPP levels.

The importance of density as a general underlying constraint to EPP might not be accepted if empirical evidence is simply assessed by counting the number of significant tests (see the criticism by Moller and Ninni 1998). Based on the number of studies published, evidence for the density hypothesis within or among populations is therefore usually cited as “not consistent” (Griffith et al. 2002), “equivocal” (Tarof et al. 1998) or “contrasting” (Charmantier and Perret 2004). Contradictory evidence can easily be found in the literature (see Appendix S2, Supporting information), even within the same species (e.g., red-winged blackbirds, Gibbs et al. 1990; Westneat and Mays 2005), and this leads to the conclusion that the influence of density on EPP is not as consistent or strong (Westneat and Stewart 2003; Neudorf 2004) as initially envisioned (Birkhead 1979; Westneat et al. 1990). This conclusion may be premature, especially when considering that some studies have been cited as not supporting the density hypothesis, even though they did not apply any tests.

Aside from local density, other factors influence variation in EPP. For example, female identity in our study always explained a significant amount of the variance in EPP in within-population analyses. Similarly, depending on the quality of their social mates, females may have different propensities to seek EPCs (Kempenaers et al. 1992), to obtain direct benefits like infertility insurance or indirect benefits like good genes or an increase in heterozygosity of their offspring (Griffith et al. 2002; Westneat and Stewart 2003). These factors contribute to variation in EPP on top of the variation that is explained by density.

## Conclusions

We showed that density explains variation in levels of EPP. Our approach to testing the density hypothesis simultaneously included analyses within and among subpopulations, which has previously been attempted only once (Krokene and Lifjeld 2000). Our results add to the list of studies that support the density hypothesis in within-population analyses and also corroborate the few studies and meta-analyses supporting an effect of density on EPP rate at the (sub)population level within species (Westneat and Sherman 1997; Moller and Ninni 1998). That factors other than density contribute to variation in EPP may explain why a general relationship between density and EPP has not been found yet in among-species comparisons (Westneat and Sherman 1997). However, it may be worthwhile reassessing the importance of density to variation in EPP rates among species using density measures that truly reflect extra-pair mating opportunities while taking confounding factors into account.

## Data accessibility

DNA sequences: Genbank accessions EF601685- EF601699.

## Supporting Information

**Appendix S1.** Breakdown of extra-pair paternity rates (EPP rate, i.e. extra-pair offspring divided by total number of offspring per brood), nearest neighbor distances (NND) and number of neighbors (NN) per subpopulation and year. Values indicate means and, in parentheses, SD. N = number of broods with offspring surviving to blood sampling.

**Appendix S2.** List of papers being supportive or non-supportive of the density hypothesis.
