# Supplementary material for: New support for an old hypothesis: density affects extra-pair paternity
Source: Ecol Evol. 2013 Feb 13;3(3):694–705. doi: 10.1002/ece3.489 (PMC3605856; doi:10.1002/ece3.489)
Supplement: Supplementary file 1 [file ece30003-0694-SD1.doc]

**Supporting information**

Appendix S1 Breakdown of extra-pair paternity rates (EPP rate, i.e. extra-pair offspring divided by total number of offspring per brood), nearest neighbor distances (NND) and number of neighbors (NN) per subpopulation and year. Values indicate means and, in parentheses, SD. N = number of broods with offspring surviving to blood sampling.

Appendix S2 List of papers being supportive or non-supportive of the density hypothesis.

Supportive

Bjornstad G, Lifjeld JT (1997) High frequency of extra-pair paternity in a dense and synchronous population of Willow warblers *Phylloscopus trochilus*. *Journal of Avian Biology*, **28**, 319-324.

Charmantier A, Perret P (2004) Manipulation of nest-box density affects extra-pair paternity in a population of blue tits (*Parus caeruleus*). *Behavioral Ecology and Sociobiology*, **56**, 360-365.

Estep LK, Mays H, Keyser AJ, Ballentine B, Hill GE (2005) Effects of breeding density and plumage coloration on mate guarding and cuckoldry in blue grosbeaks (Passerina caerulea). *Canadian Journal of Zoology-Revue Canadienne De Zoologie*, **83**, 1143-1148.

Gibbs HL, Weatherhead PJ, Boag PT*, et al.* (1990) Realized reproductive success of polygynous Red-Winged Blackbirds revealed by DNA markers. *Science*, **250**, 1394-1397.

Gowaty PA, Bridges WC (1991) Nestbox availability affects extra-pair fertilizations and conspecific nest parasitism in Eastern Bluebirds, *Sialia sialis*. *Animal Behaviour*, **41**, 661-675.

Gray EM (1996) Female control of offspring paternity in a western population of red-winged blackbirds (Agelaius phoeniceus). *Behavioral Ecology and Sociobiology*, **38**, 267-278.

Hasselquist D, Bensch S, Vonschantz T (1995) Low frequency of extrapair paternity in the polygynous Great Reed Warbler, *Acrocephalus arundinaceus*. *Behavioral Ecology*, **6**, 27-38.

Hoi H, Hoi-Leitner M (1997) An alternative route to coloniality in the bearded tit: females pursue extra-pair fertilizations. *Behavioral Ecology*, **8**, 113-119.

Krokene C, Lifjeld JT (2000) Variation in the frequency of extra-pair paternity in birds: a comparison of an island and a mainland population of blue tits. *Behaviour*, **137**, 1317-1330.

Langefors A, Hasselquist D, von Schantz T (1998) Extra-pair fertilizations in the sedge warbler. *Journal of Avian Biology*, **29**, 134-144.

Lindstedt ER, Oh KP, Badyaev AV (2007) Ecological, social, and genetic contingency of extrapair behavior in a socially monogamous bird. *Journal of Avian Biology*, **38**, 214-223.

Moller AP (1991) Density-dependent extra-pair copulations in the swallow *Hirundo rustica*. Ethology, **87**, 316-329.

Moller AP, Ninni P (1998) Sperm competition and sexual selection: a meta-analysis of paternity studies of birds. *Behavioral Ecology and Sociobiology*, **43**, 345 - 358.

Richardson DS, Burke T (1999) Extra-pair paternity in relation to male age in Bullock's orioles. *Molecular Ecology*, **8**, 2115-2126.

Ryder TB, Fleischer RC, Shriver WG, Marra PP (2012) The ecological–evolutionary interplay: density-dependent sexual selection in a migratory songbird. *Ecology and Evolution*, **2**, 976-987.

Stewart SLM, Westneat DF, Ritchison G (2010) Extra-pair paternity in eastern bluebirds: effects of manipulated density and natural patterns of breeding synchrony. *Behavioral Ecology and Sociobiology,* **64**, 463-473.

Westneat DF, Sherman PW (1997) Density and extra-pair fertilizations in birds: a comparative analysis. *Behavioral Ecology and Sociobiology*, **41**, 205-215.

Yezerinac SM, Gibbs HL, Briskie JV, Whittam R, Montgomerie R (1999) Extrapair paternity in a far northern population of yellow warblers *Dendroica petechia*. *Journal of Avian Biology*, **30**, 234-237.

Non-supportive

Ardern SL, Ma W, Ewen JG, Armstrong DP, Lambert DM (1997) Social and sexual monogamy in translocated New Zealand robin populations detected using minisatellite DNA. *Auk*, **114**, 120-126.

Barber CA, Robertson RJ, Boag PT (1996) The high frequency of extra pair paternity in tree swallows is not an artifact of nestboxes. *Behavioral Ecology and Sociobiology*, **38**, 425-430.

Bollinger EK, Gavin TA (1991) Patterns of extra-pair fertilizations in Bobolinks. *Behavioral Ecology and Sociobiology*, **29**, 1-7.

Bouwman KM, Komdeur J (2006) Weather conditions affect levels of extra-pair paternity in the reed bunting *Emberiza schoeniclus*. *Journal of Avian Biology*, **37**, 238-244.

Charmantier A, Blondel J (2003) A contrast in extra-pair paternity levels on mainland and island populations of mediterranean blue tits. *Ethology*, **109**, 351-363.

Casey, AE, Sandercock BK, Wisely, SM (2011) Genetic parentage and local population structure in the socially monogamous upland sandpiper. Condor, **113**, 119-128.

Chuang HC, Webster MS, Holmes RT (1999) Extrapair paternity and local synchrony in the Black-throated Blue Warbler. *Auk*, **116**, 726-736.

Dunn PO, Robertson RJ, Michaud-Freeman D, Boag PT (1994a) Extra-pair paternity in tree swallows: why do females mate with more than one male? *Behavioral Ecology and Sociobiology*, **35**, 273-281.

Dunn PO, Whittingham LA, Lifjeld JT, Robertson RJ, Boag PT (1994b) Effects of breeding density, synchrony, and experience on extrapair paternity in tree swallows. *Behavioral Ecology*, **5**, 123-129.

Griffith SC, Stewart IRK, Dawson DA, Owens IPF, Burke T (1999) Contrasting levels of extra-pair paternity in mainland and island populations of the house sparrow (*Passer domesticus*): is there an 'island effect'? *Biological Journal of the Linnean Society*, **68**, 303-316.

Gullberg A, Tegelstrom H, Gelter HP (1992) DNA fingerprinting reveals multiple paternity in families of Great and Blue Tits (*Parus major* and *Parus caeruleus*). *Hereditas*, **117**, 103-108.

Gyllensten UB, Jakobsson S, Temrin H (1990) No evidence for illegitimate young in monogamous and polygynous warblers. *Nature*, **343**, 168-170.

Hill GE, Montgomerie R, Roeder C, Boag P (1994) Sexual selection and cuckoldry in a monogamous songbird: implications for sexual selection theory. *Behavioral Ecology and Sociobiology*, **35**, 193-199.

Johannessen LE, Slagsvold T, Hansen BT, Lifjeld JT (2005) Manipulation of male quality in wild tits: effects on paternity loss. *Behavioral Ecology*, **16**, 747-754.

Korpimaki E, Lahti K, May CA*, et al.* (1996) Copulatory behaviour and paternity determined by DNA fingerprinting in kestrels: Effects of cyclic food abundance. *Animal Behaviour*, **51**, 945-955.

Leisler B, Beier J, Staudter H, Wink M (2000) Variation in extra-pair paternity in the polygynous great reed warbler (*Acrocephalus arundinaceus*). *Journal für Ornithologie*, **141**, 77-84.

Moore OR, Stutchbury BJM, Quinn JS (1999) Extrapair mating system of an asynchronously breeding tropical songbird: the mangrove swallow. *Auk*, **116**, 1039-1046.

Moore JA., Kamarainen AM, Scribner KT, Mykut C, Prince HH (2012) The effects of anthropogenic alteration of nesting habitat on rates of extra-pair fertilization and intraspecific brood parasitism in Canada geese *Branta canadensis*. Ibis, **154**, 354-362.

Stutchbury BJM, Quinn JS (1999) Extrapair mating system of an asynchronously breeding tropical songbird: the mangrove swallow. *Auk*, **116**, 1039-1046.

Olsen BJ, Greenberg R, Fleischer RC, Walters JR (2008) Extrapair paternity in the swamp sparrow, *Melospiza georgiana*: male access or female preference? Behavioral Ecology and Sociobiology, **63**,285-294.

Orell M, Rytkonen S, Launonen V*, et al.* (1997) Low frequency extra-pair paternity in the willow tit *Parus montanus* as revealed by DNA fingerprinting. *Ibis*, **139**, 562-566.

Rätti O, Lundberg A, Tegelstrom H, Alatalo RV (2001) No evidence for effects of breeding density and male removal on extrapair paternity in the pied flycatcher. *Auk*, **118**, 147-155.

Reyer HU, Bollmann K, Schlapfer AR, Schymainda A, Klecack G (1997) Ecological determinants of extrapair fertilizations and egg dumping in Alpine water pipits (Anthus spinoletta). Behavioral Ecology, 8, 534-543.

Rowe KMC, Weatherhead PJ (2007) Social and ecological factors affecting paternity allocation in American robins with overlapping broods. *Behavioral Ecology and Sociobiology*, **61**, 1283-1291.

Sundberg J, Dixon A (1996) Old, colourful male yellowhammers, *Emberiza citrinella*, benefit from extra-pair copulations. *Animal Behaviour*, **52**, 113-122.

Tarof SA, Stuchbury BJ, Piper WH, Fleischer RC (1998) Does breeding density covary with extra-pair fertilizations in hooded warblers? *Journal of Avian Biology*, **29**, 145-154.

Thusius KJ, Dunn PO, Peterson KA, Whittingham LA (2001) Extrapair paternity is influenced by breeding synchrony and density in the common yellowthroat. *Behavioral Ecology*, **12**, 633-639.

Vaclav R, Hoi H (2002) Importance of colony size and breeding synchrony on behaviour, reproductive success and paternity in house sparrows *Passer domesticus*. *Folia Zoologica*, **51**, 35-48.

Westneat DF, Mays HL (2005) Tests of spatial and temporal factors influencing extra-pair paternity in red-winged blackbirds. *Molecular Ecology*, **14**, 2155-2167.
